# A developed expression of chemical potential for fast deformation in nanoparticle electrodes of lithium-ion batteries

**DOI:** 10.1186/s11671-019-3094-8

**Published:** 2019-08-01

**Authors:** Feng Wang, Kai Zhang, Bailin Zheng

**Affiliations:** School of Aerospace Engineering and Applied Mechanics, Tongji University, Shanghai, 200092 China

**Keywords:** Chemical potential, Fast deformation, Diffusion-induced stress, Lithium-ion batteries

## Abstract

In this paper, we propose a developed expression of chemical potential without the assumption of low deformation rate to account for the diffusion induced stress and the distribution of Li concentration in nanoparticle electrodes of lithium-ion batteries. The difference between the developed and traditional expressions on the stress evolution in a spherical nanoparticle electrode made of silicon is analyzed under both potentiostatic and galvanostatic operations, using the derived diffusion equation and the finite deformation theory. The numerical result suggests that the difference between these two expressions of chemical potential is significant under potentiostatic operation, rather than that under galvanostatic operation. A critical radius, where there is no difference between the Li flux caused by these two expressions of chemical potential as well as the Cauchy hydrostatic stress during most of the lithiated process, is firstly reported in this work.

## Introduction

For the development of portable electronic devices, electric vehicles and large-scale energy storage, a number of high-capacity electrode materials such as silicon, which experience extreme changes in volume during lithiation, are proposed to be applied in lithium batteries [1–3]. The stress-induced by homogeneous volumetric deformation is called diffusion induced stress, which may induce brittle fracture during cyclic charge and discharge, and this negative effect further degrades the capacity of the battery [4]. Composite materials of lithium-ion battery electrodes are generally complex, and their morphologies are different, which makes it more difficult to explain battery behavior by theory or equation. In theoretical models, the properties of composite materials are usually simulated by considering the changes of electrode material parameters in space coordinates, while the interface effect of composite materials is ignored. At present, three typical electrode shapes, i.e., spherical, cylindrical and plate, are mainly considered in the theoretical model. Among them, spherical and cylindrical shapes are usually one-dimensional models, and both one-dimensional and two-dimensional models for plate electrodes are available. Recently, a number of studies have focused on the diffusion-induced stress in silicon nanoparticle electrodes of lithium-ion batteries. For example, Yang et al. [5] presented a chemo-mechanical model to investigate the lithiation-induced phase transformation, morphological evolution, stress generation and fracture in crystalline silicon nanowires. Li et al. [6] studied the effect of local velocity on diffusion-induced stress in silicon nanoparticle electrodes. Zhao et al. [7] analyzed the diffusion induced stress with the consideration of inelastic deformation of host material. In all these aforementioned works, the fundamental physics involved is the atomic or ionic diffusion in solids under multiple driving forces. Atomic diffusion in a solid may change the solid composition from its stoichiometric state and be affected by the diffusion induced stress. Such stress and diffusion interaction is governed by the thermodynamic equilibrium of the solids.

Larche and Cahn [8] developed a thermodynamic framework for multi-component solids which reach equilibrium under non-hydrostatic stress. The framework was based on the assumption that the deformation caused by composition change is small and isotropic. As a result, a stress-dependent chemical potential was introduced to account for the interaction between stress and diffusion. Wu [9] derived a different stress-dependent chemical potential in which the Eshelby momentum tensor is involved instead of the hydrostatic Cauchy stress. On this basis, Cui et al.[10] have proposed a new chemical potential for the finite deformation of solids. However, in these works, the derivation is only to be rigorous when the deformation is small or the deformation rate is low enough compared with diffusion. It is likely to make a significant error for a silicon electrode because of its large compositional volumetric expansion (∼ 400%) when it is lithiated rapidly.

In this paper, we present a developed expression of chemical potential without the assumption of low deformation rate, distinguished from the traditional expression of Cui [10]. This model is established for the fast deformation of the electrodes during charging or discharging and is independent of the morphologies because chemical potential is an intensive quantity rather than an extensive quantity. The difference between these two expressions of chemical potential on the distributions of stress and Li-concentration are analyzed under both potentiostatic and galvanostatic operations in Si nanoparticle electrodes. The result reveals that the difference increases with the increase of deformation rate. A critical radius, where there is no difference between the Li flux caused by these two expressions of chemical potential as well as the Cauchy hydrostatic stress during most of the lithiated process, is found at the same time.

## Methods

### Mechanical Equations

The insertion of lithium into electrodes can cause volumetric change. For convenience, we employ two ways to describe the deformation and motion of the solids, namely, the Lagrangian description and the Eulerian description. The motion of material particles in a continuum medium can be described by

1 $$ \mathbf{U}=\mathbf{x}-\mathbf{X} $$

where **x** is the Euler coordinates, **X** is the Lagrange coordinates, and **U** is the displacement field. The change in the shape of the continuum solid can be characterized by the deformation gradient tensor, given by

2 $$ \mathbf{F}=\frac{\partial \mathbf{x}}{\partial \mathbf{X}}=\mathbf{I}+\mathrm{Grad}\mathbf{U}, $$

where Grad represents the gradient operator in the Lagrangian description, and **I** is the unit tensor of second order.

For a spherical particle, the Lagrange coordinates and the Euler coordinates of a material point are (R, Θ, Φ) and (r, θ, φ) respectively in the spherical system. Then, the deformation gradient tensor **F** is found as

3 $$ \mathbf{F}=\left[\begin{array}{ccc}{F}_R& 0& 0\\ {}0& {F}_{\Theta}& 0\\ {}0& 0& {F}_{\Phi}\end{array}\right]=\left[\begin{array}{ccc}1+\partial u/\partial R& 0& 0\\ {}0& 1+u/R& 0\\ {}0& 0& 1+u/R\end{array}\right]. $$

During charging or discharging, the shape change of the electrode can be divided into two processes: (a) a shape change due to the insert of lithium and (b) a reversible elastic deformation. These two deformation processes can be described by two separated gradient tensors and the total deformation gradient tensor can be written as

4 $$ \mathbf{F}={\mathbf{F}}^e{\mathbf{F}}^c $$

where **F**^*e*^ represents the elastic deformation, **F**^*c*^ represents the shape change due to the insert of lithium. Equation (4) represents the process of electrode material from the initial (undeformed) state to the current (deformed) state. Assuming the shape change due to the insert of lithium is isotropic, **F**^*c*^ can be given by

5 $$ {\mathbf{F}}^c={\left(1+\Omega C\right)}^{1/3}\mathbf{I}, $$

where Ω represents the partial molar volume.

From Eq. (3–5), the elastic deformation gradient tensor **F**^*e*^ is

6 $$ {\mathbf{F}}^e={\left(1+\Omega (R)C\right)}^{-1/3}\left[\begin{array}{ccc}1+\partial u/\partial R& 0& 0\\ {}0& 1+u/R& 0\\ {}0& 0& 1+u/R\end{array}\right]. $$

The total Green-Lagrange strain tensor **E** can be written as

7 $$ \mathbf{E}=\frac{1}{2}\left({\mathbf{F}}^T\mathbf{F}-\mathbf{I}\right), $$

where the elastic strain tensor **E**^e^ and diffusion-induced strain tensor **E**^*c*^ are

8 $$ {\mathbf{E}}^e=\frac{1}{2}\left({\left({\mathbf{F}}^e\right)}^T{\mathbf{F}}^e-\mathbf{I}\right),{\mathbf{E}}^c=\frac{1}{2}\left({\left({\mathbf{F}}^c\right)}^T{\mathbf{F}}^c-\mathbf{I}\right), $$

respectively.

Substituting Eq. (6) into Eq. (8), the radial and tangential components of the Green-Lagrange strain tensor are

9 $$ {E}_R^e=\frac{1}{2}\left[\frac{{\left(1+\partial u/\partial R\right)}^2}{{\left(1+\Omega (R)C\right)}^{2/3}}-1\right], $$

10 $$ {E}_{\Theta}^e={E}_{\Phi}^e=\frac{1}{2}\left[\frac{{\left(1+u/R\right)}^2}{{\left(1+\Omega (R)C\right)}^{2/3}}-1\right]. $$

The constitutive relation for the deformation can be determined from the strain energy density as

11 $$ \mathbf{P}=\frac{\partial W}{\partial \mathbf{F}}=\frac{\partial W}{\partial {\mathbf{E}}^e}\frac{\partial {\mathbf{E}}^e}{\partial {\mathbf{F}}^e}\frac{\partial {\mathbf{F}}^e}{\partial \mathbf{F}}, $$

where *W* is the elastic strain energy density in the Lagrangian description, and **P** is the first Piola-Kirchhoff stress. Furthermore, if the material is linearly elastic, *W* can be written as a quadratic function of the Green-Lagrange strain tensor

12 $$ W=\frac{1}{2}{\mathbf{E}}^e:\mathbf{C}:{\mathbf{E}}^e=\det \left({\mathbf{F}}^c\right)\frac{E_h}{2\left(1+\nu \right)}\left[\frac{\nu }{\left(1-2\nu \right)}{\left[ tr\left({\mathbf{E}}^e\right)\right]}^2+ tr\left({\mathbf{E}}^e{\mathbf{E}}^e\right)\right]. $$

Here, *E*_*h*_ and *ν* are Young’s modulus and Poisson’s ratio, respectively, **C** is the stiffness tensor, and det(**F**^*c*^) is the determinant of the deformation gradient tensor for the diffusion-induced deformation.

Finally, the first Piola-Kirchhoff stress is given by

13 $$ \mathbf{P}=\det \left({\mathbf{F}}^c\right)\frac{E_h}{\left(1+\nu \right)}\left[\frac{\nu }{\left(1-2\nu \right)} tr\left({\mathbf{E}}^e\right)+{\mathbf{E}}^e\right]{\mathbf{F}}^e{\left({\mathbf{F}}^c\right)}^{-1}. $$

Combining Eqs. (5), (9), (10), and (13), the corresponding components of the first Piola-Kirchhoff (P-K) stress tensor are

14 $$ {P}_R={\left(1+\Omega C\right)}^{1/3}\frac{E_h}{2\left(1+\nu \right)\left(1-2\nu \right)}\left(1+\frac{\partial u}{\partial R}\right)\left[\left(1-v\right){E}_R^e+2{vE}_{\Theta}^e\right], $$

15 $$ {P}_{\Theta}={P}_{\Phi}={\left(1+\Omega C\right)}^{1/3}\frac{E_h}{2\left(1+\nu \right)\left(1-2\nu \right)}\left(1+\frac{u}{R}\right)\left({vE}_R^e+{E}_{\Theta}^e\right), $$

And the first P-K stress must satisfy the condition of equilibrium in the absence of body force

16 $$ \frac{\partial {P}_R}{\partial R}+2\frac{P_R-{P}_{\Phi}}{R}=0, $$

with initial and boundary conditions

17 $$ u\left(0,\mathrm{t}\right)=0,{P}_R\left({\mathrm{R}}_0,\mathrm{t}\right)=0. $$

### Mass Transport Equation

The concentration and the diffusion flux of lithium in electrodes, which are functions of coordinates and time, will be called *C*(**X**, t) and **J**(**X**, t) in the Lagrangian description, and they should be satisfied with the mass transport equation written as

18 $$ \frac{\partial C}{\partial t}+\mathrm{Div}\mathbf{J}=0, $$

where Div represents the divergence operator in the Lagrangian description. Considering the characteristic of spherical symmetry, diffusion occurs only in the radial direction and we use *J*(*R*, t) to represent the radial component of **J**(**X**, t). In the spherical system, Eq. (18) becomes

19 $$ \frac{\partial C\left(R,t\right)}{\partial t}+\frac{\partial \left({R}^2J\left(R,t\right)\right)}{R^2\partial R}=0. $$

The diffusion of lithium in electrodes is driven by chemical potential gradient, and the radial flux *J*(*R*, t) is proportional to the gradient of chemical potential *μ*, as [11]

20 $$ J=-\frac{CD}{R_g{TF}_{11}{F}_{11}}\frac{\partial \mu }{\partial R}, $$

where *D* is the diffusivity, *R*_*g*_ is the gas constant, and *T* is the temperature. *μ* is defined as the deviation of total energy density to the concentration and can be written as

21 $$ \mu =\frac{\mathrm{\partial \Pi }}{\partial C}. $$

Assume that the energy density of the system, Π, can be described as the sum of chemical energy density and strain energy density. So, the total internal energy density can be written as

22 $$ \Pi \left(\mathbf{X},\mathrm{t}\right)=\varphi (C)+W\left(C,{\mathbf{E}}^e\right). $$

Substituting Eq. (22) into Eq.(21), the chemical potential *μ* can be shown to be

23 $$ \mu =\frac{\mathrm{\partial \Pi }}{\partial C}=\frac{\partial \varphi }{\partial C}+\frac{\partial W}{\partial C}={\mu}_0(C)+\tau \left({\mathbf{E}}^e,C\right) $$

where *μ*_0_(*C*) and *τ*(**E**^*e*^, *C*) are the stress-independent and stress-dependent parts of the chemical potential respectively. And

24 $$ {\mu}_0(C)={\mu}_0+{R}_gT\ln \left(\gamma C\right) $$

where *μ*_0_ is a constant that represents the chemical potential at a standard state, and *γ* is the activity coefficient which represents the effects of interactions among the atoms/molecules. For a dilute solution, interactions among the atoms/molecules are negligible; thus, *γ* = 1.

We focus on the stress-dependent part of the chemical potential *τ*(**E**^*e*^, C), which is the derivative of strain energy density *W* with respect to the concentration of lithium *C.* Traditionally, Π(**X**, t) is considered to be Helmholtz free energy density and therefore this step is carried out for fixed deformation written as [11]

25 $$ {\tau}_H\left({\mathbf{E}}^e,\mathrm{C}\right)=\frac{\partial W}{\partial C}\left|\begin{array}{c}\\ {}\mathbf{F}\end{array}\right.=-\det \left({\mathbf{F}}^e\right){\sigma}_m\Omega . $$

The subscript *H* means that it is caused by the Helmholtz free energy density. The chemical potential turns out to be

26 $$ \mu ={\mu}_0+{R}_gT\kern0.5em \ln (C)-\det \left({\mathbf{F}}^e\right)\Omega {\sigma}_m $$

where *σ*_*m*_ is the Cauchy hydrostatic stress in Eulerian description and can be obtained by

27 $$ {\boldsymbol{\upsigma}}_m=\frac{1}{3} tr\left(\boldsymbol{\upsigma} \right)=\frac{1}{3} tr\left({\det}^{-1}\left(\mathbf{F}\right){\mathbf{PF}}^T\right). $$

It is worth to note that the stiffness **C** of the electrode material is assumed to be independent of the concentration of lithium *C* in Eq. (12). In addition, det(**F**^*e*^) ≈ 1 is widely accepted so that it is ignored usually. In the rest of this paper, we call Eq. (26) as the traditional expression of chemical potential. On the other hand, Π(**X**, t) is considered to be the Gibbs free energy density in some studies [12, 13] on phase field model so that we cannot get a developed expression of *τ*(**E**^*e*^, C), and

28 $$ {\tau}_G\left({\mathbf{E}}^e,\mathrm{C}\right)=\frac{\partial W}{\partial C}. $$

The subscript *G* means that it is caused by the Gibbs free energy density. In this case, *μ*becomes

29 $$ \mu ={\mu}_0+{R}_gT\kern0.5em \ln (C)-\frac{\partial W}{\partial C} $$

and we call Eq. (29) as the developed expression of chemical potential.

The mass transport equation is consisted by Eqs. (19), (20), (26), and (29) with traditional and developed expressions of chemical potential. In the remaining part of this paper, we will compare the effects of these two expressions of chemical potential on the diffusion induced stress and Li concentration under different charging methods.

In thermodynamics, the Helmholtz free energy is a thermodynamic potential that measures the useful work obtainable from a closed thermodynamic system at a constant temperature and volume. In contrast, the Gibbs free energy measures the maximum of reversible work that may be performed by a thermodynamic system at a constant temperature and pressure. In solids with low stress level, the Gibbs free energy is approximately equivalent to the Helmholtz free energy, because the deformation of solids is small usually. This assumption is suitable for most solid materials due to their small diffusion induced deformation, but except for silicon because of its large volumetric expansion during lithiation. In fact, diffusion and deformation take place at the same time, so that it is not suitable assuming that there is no deformation happening while the concentration is changing. Even so, as can be seen from Eq. (25), the traditional expression of chemical potential is still accurate when the deformation rate is low enough. However, it is likely to cause great errors when a Si nanoparticle electrode is lithiated rapidly.

The electrode is lithiated with a constant lithium-ion concentration on its surface, namely the potentiostatic operation, or with a constant flux on its surface, namely the galvanostatic operation. The boundary conditions of Eq. (19) are

30 $$ C\left({R}_0,\mathrm{t}\right)={C}_{\mathrm{max}},\kern0.5em \mathrm{for}\ \mathrm{t}\ge 0, $$

31 $$ J\left({R}_0,\mathrm{t}\right)={j}_0{\left(1+u/R\right)}^2,\kern0.5em \mathrm{for}\ \mathrm{t}\ge 0, $$

respectively. The initial condition is written as

32 $$ C\left(R,0\right)=0\ \mathrm{for}\ 0\le R\le {R}_0, $$

for each charge operation. Here, *C*_max_ is the maximum lithium concentration of the material and *j*_0_ is a constant representing the charge current.

### Numerical Implementation

It is very difficult to obtain the analytical solution of the above system consisting of partial differential equations, if not impossible. With Eqs. (1)–(3) and (13)–(18), we calculate the evolution of diffusion-induced stress and lithium concentration numerically by using the COMSOL multiphysics software. The lithiation of a silicon nano-electrode under both potentiostatic and galvanostatic operations are studied, with different expressions of chemical potential. The material properties of Si and operating parameters used in the simulation are listed in Table 1. For convenience, the corresponding dimensionless substitution of coordinate, stress, and concentration are used in the figures.

**Table 1:** Material properties and operating parameters

Parameter	Value	Unit	Source
*D*	1 × 10^−16^	*m*^2^/*s*	Ref. [14]
*ν*	0.28	/	Ref. [14]
*Ω*	8.18 × 10^−6^	*m*^3^/*mol*	Ref. [15]
*E*	9 × 10^10^	*Pa*	Ref. [14]
*R*_*g*_	8.31	*J*/(*mol* ⋅ *K*)	Ref. [16]
*T*	273	*K*	Ref. [16]
*j*_0_	3 × 10^−3^	*mol*/(*m*^2^ · *s*)	Assumed
*R*_0_	2.5 × 10^−7^	*m*	Assumed
*C*_max_	3.67 × 10^5^	*mol*/*m*^3^	Ref. [16]

To investigate the difference between the different expressions of chemical potential at different times in the spherical Si electrode, the state of charge (SOC) is calculated as

33 $$ SOC=\frac{\int_0^{R_0}C\left(R,t\right){R}^2 dR}{\int_0^{R_0}{C}_{\mathrm{max}}{R}^2 dR}. $$

The stress-induced diffusion fluxes in the Lagrangian description are described as

34 $$ {J}_H=\frac{\partial {\tau}_H\left({\mathbf{E}}^e,C\right)}{\partial R},{J}_G=\frac{\partial {\tau}_G\left({\mathbf{E}}^e,C\right)}{\partial R}, $$

representing the flux caused by different chemical potential expressions, respectively.

## Results and Discussion

**Fig. 1:**
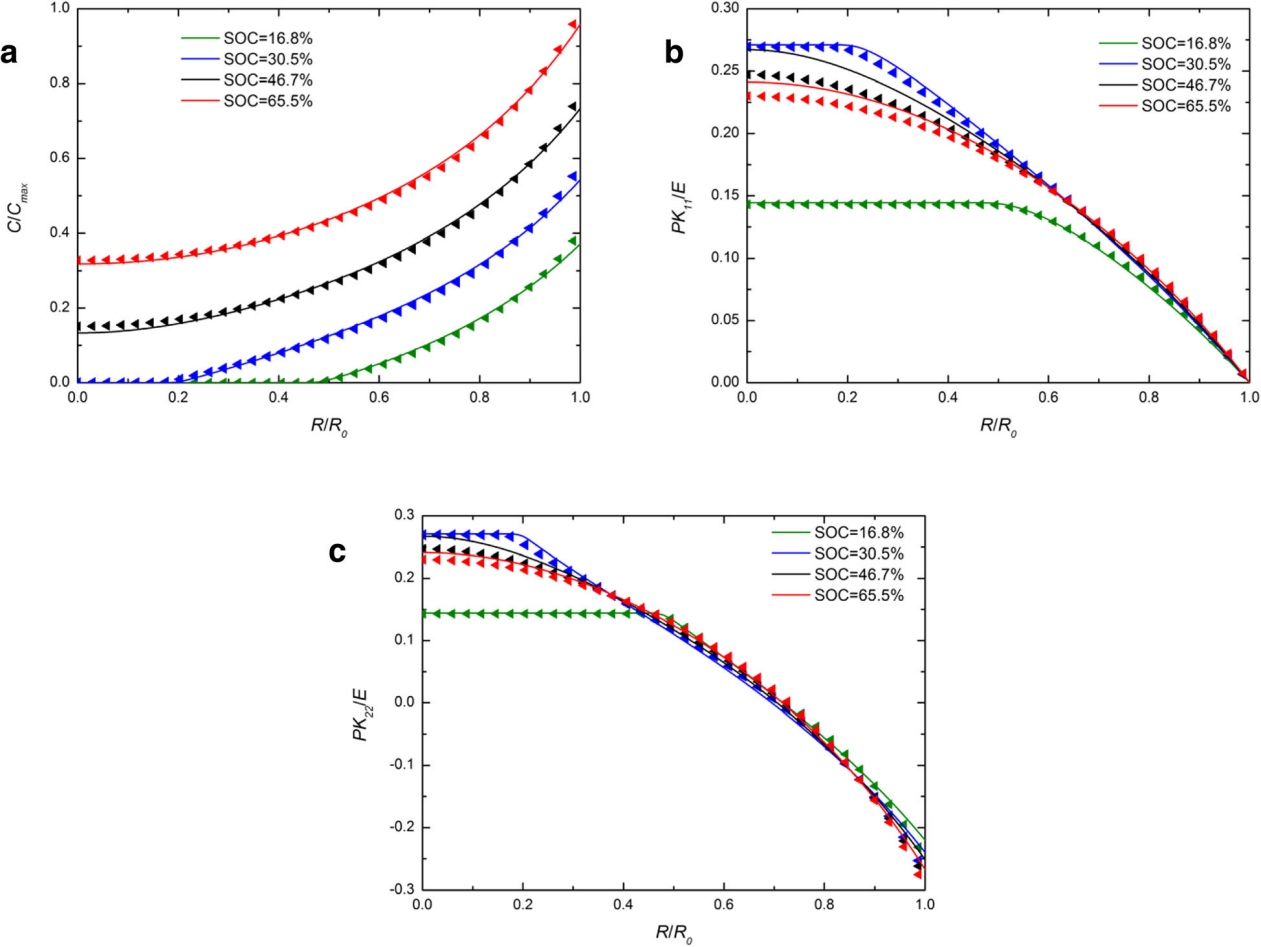
Spatial distribution of (**a**) the concentration of lithium, (**b**) radial stress, and (**c**) hoop stress in a spherical Si electrode at different SOCs under galvanostatic operation (solid lines represent the results with the traditional expression of chemical potential; triangles lines represent the results with the developed expression of chemical potential)

**Fig. 2:**
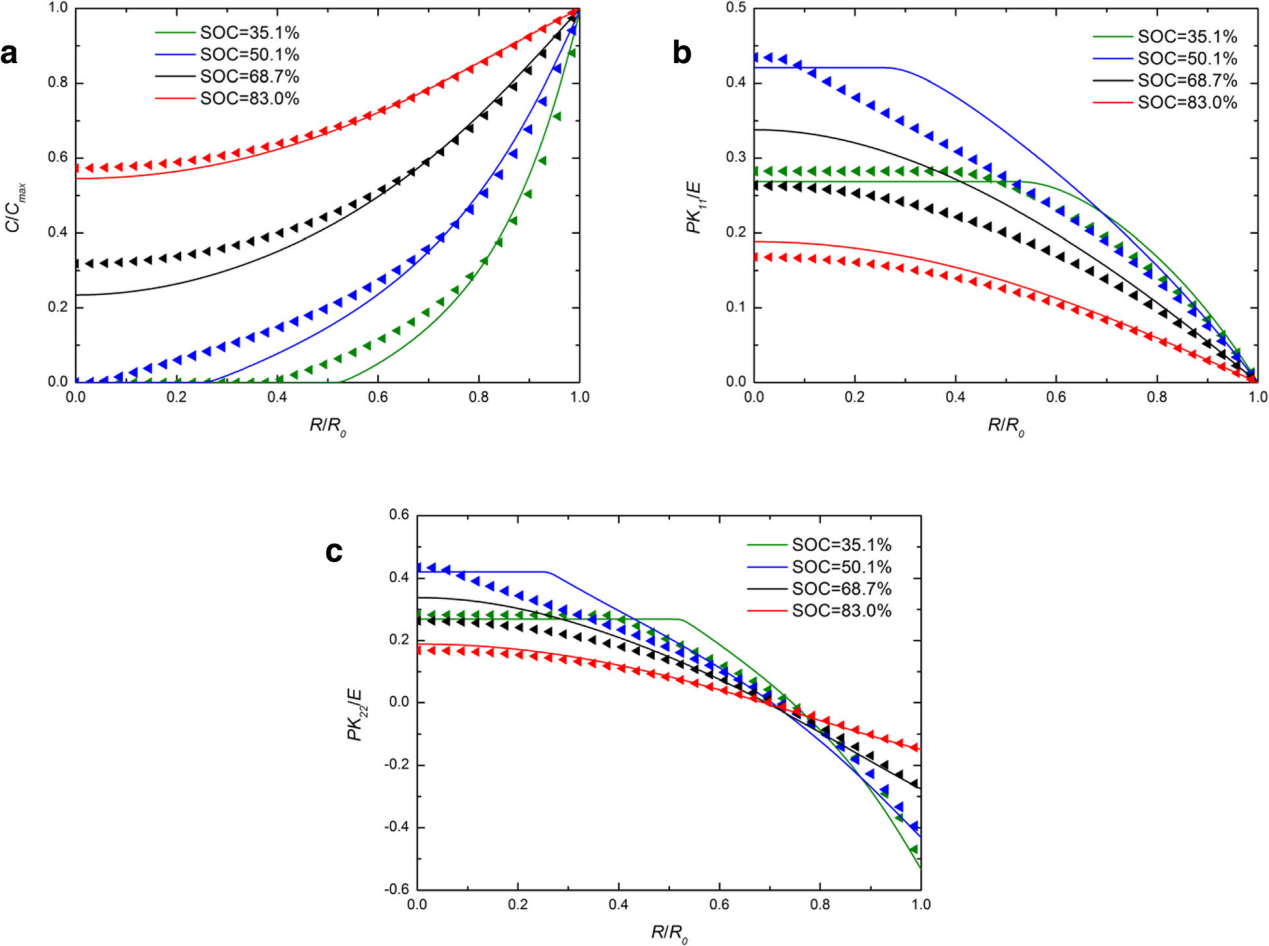
Spatial distribution of (**a**) the concentration of lithium, (**b**) radial stress, and (**c**) hoop stress in a spherical Si nanoparticle electrode at different SOCs under potentiostatic operation (solid lines represent the results with the traditional expression of chemical potential; triangles lines represent the results with the developed expression of chemical potential)

**Fig. 3:**
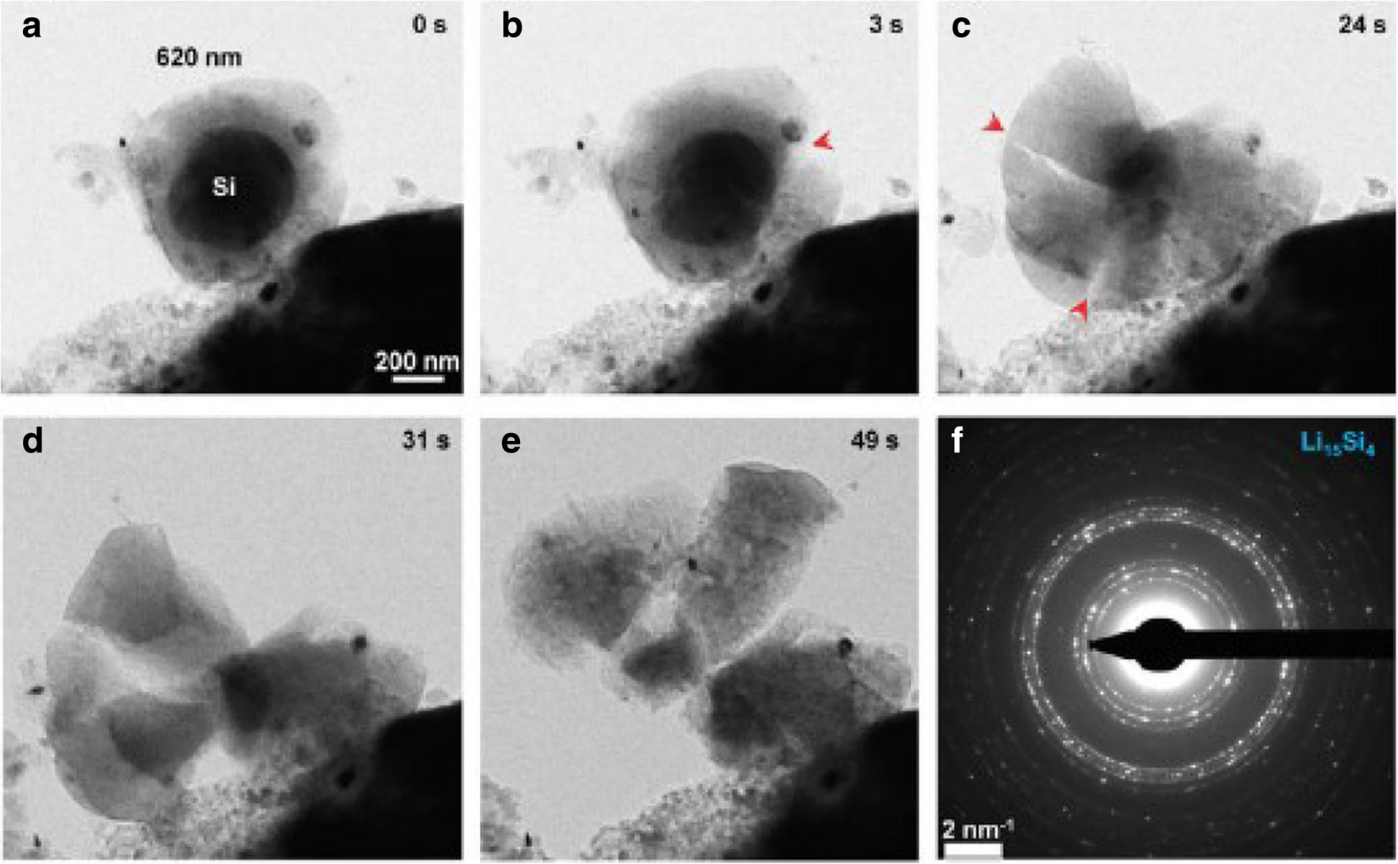
Fast deformation of a free-standing 620-nm SiNP during chemical lithiation in 1 min. **a**–**e** Time sequence of crack initiation and growth. **f** EDP indicating the formation of polycrystalline Li_15_Si_4_ as the fully lithiated phase

Figure 1 shows the spatial distribution of the concentration of lithium, radial stress, and hoop stress in a spherical Si electrode under galvanostatic operation at several SOCs. For comparison, the numerical results with both the developed and traditional expressions of chemical potential are included and they are represented by solid lines and triangles symbols, respectively. For each SOC in Fig. 1a, the solid line nearly overlaps the triangle symbols. The effect caused by the different expressions of chemical potential on the concentration of lithium can be ignored. In Fig. 1b and Fig. 1c, for the SOCs of 46.7% and 65.5%, the solid lines are higher than the triangles in the center, while they almost overlap outside, just like that at other SOCs. On the whole, there is a slight effect on the concentration of lithium and stresses under galvanostatic operation. Figure 2 shows the spatial distributions of the concentration of lithium, radial stress, and hoop stress in a spherical Si electrode under potentiostatic operation at several SOCs. It is worth mentioning that the radial and hoop stresses first increase and then decrease with the increase of SOC in both Fig. 1 and Fig. 2. It is because that the silicon electrode at the initial state or fully lithiated state is stress-free, since there is no concentration gradient. Compared with Fig. 1a, the difference between solid lines and triangles is greater in Fig. 2a. Due to the lithium concentration on the surface is a constant *C*_max_ under potentiostatic operation, the charge rate is higher than the deformation rate that of galvanostatic operation and the deformation rate as well. However, the total deformation under the same SOC is almost the same regardless of the charge method, just taking different time. It indicates that the influence on the distribution of lithium caused by different expressions of chemical potential is only related to the deformation rate rather than the deformation and increases with the increase of the deformation rate. In fact, existing experiments show that silicon electrodes deform very quickly during lithiation under certain charging modes. As we can see from Fig. 3 [17], the Si anode is fully deformed in 1 min with a 2-V potential with respect to the Li metal. In this condition, the results solved by these two expressions of chemical potential will be significantly different. Unfortunately, in this case, the stress of the electrode cannot be measured accurately and therefore cannot be quantitatively compared with our model.

**Fig. 4:**
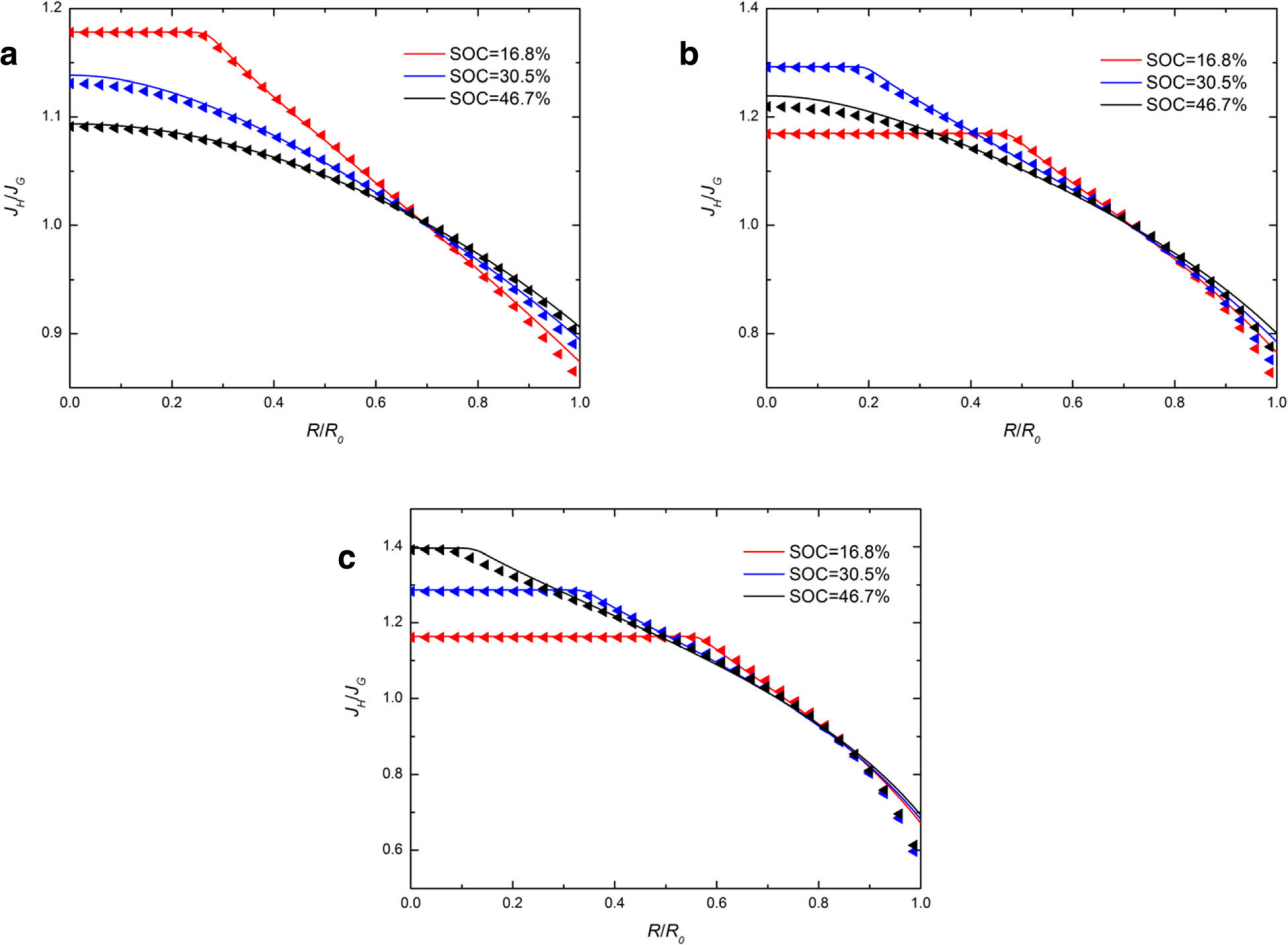
Spatial distribution of *J*_*H*_/*J*_*G*_ in a spherical Si nanoparticle electrode at different SOCs under galvanostatic operation with (**a**) *j* = 0.5*j*_0_, (**b**) *j* = *j*_0_, and (**c**) *j* = 1.5*j*_0_ (solid lines represent the results with the traditional expression of chemical potential; triangles lines represent the results with the developed expression of chemical potential)

Figure 4 shows the spatial distribution of *J*_*H*_/*J*_*G*_ in a spherical Si electrode at different SOCs under galvanostatic operation with different *j*. In Fig. 4, the solid lines almost coincide with the triangles, which indicates that different chemical potential expressions have a slight effect on the ratio of *J*_*H*_ and *J*_*G*_. It is evident that the range of the values of *J*_*H*_/*J*_*G*_ increases with the increase of the charge current. This is because the larger charge current leads to higher deformation rates and therefore causing the greater impact of different expressions of chemical potential. The ratio is always greater than 1 in the center and less than 1 on the surface. It suggests that the flux obtained from the developed expression of chemical potential on the surface is larger than that obtained from the traditional expression, while the opposite is true in the center. We notice that all the solid lines and triangles in Fig. 4 nearly intersect with one point. In addition, the ratio corresponding to the intersection is always about 1 no matter which current the electrode is charged with. It suggests that there is a critical radius where the flux is less affected by the different chemical potential expressions. We call it the chemical potential independent region (CIR). Obviously, CIR is always near the surface of the spherical electrode and is closer to the surface as the charge current increases.

**Fig. 5:**
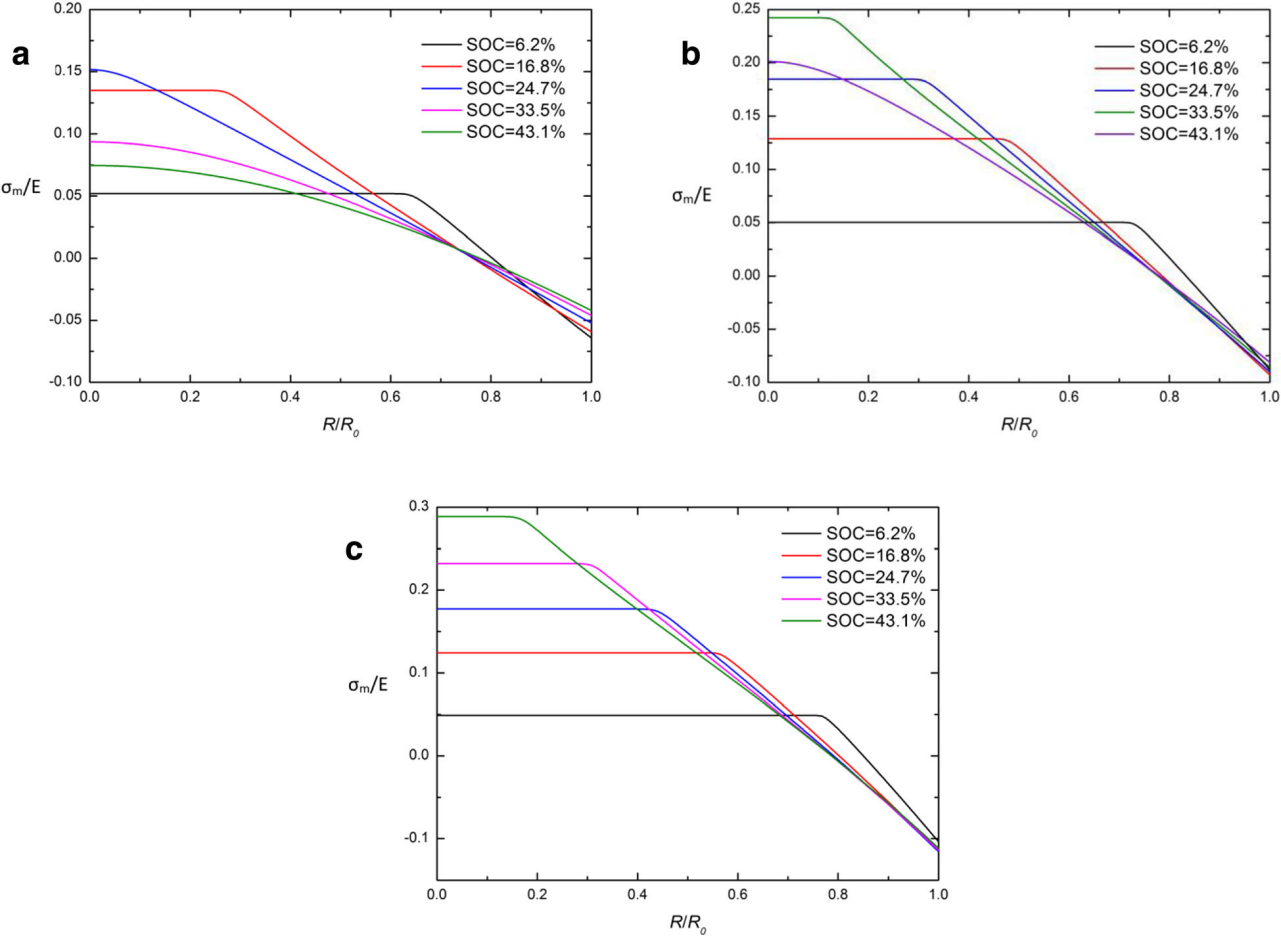
Spatial distribution of *σ*_m_/E in a spherical Si nanoparticle electrode at different SOCs under galvanostatic operation with the traditional expression of chemical potential and (**a**) *j* = 0.5*j*_0_, (**b**) *j* = *j*_0,_ and (**c**) *j* = 1.5*j*_0_

**Fig. 6:**
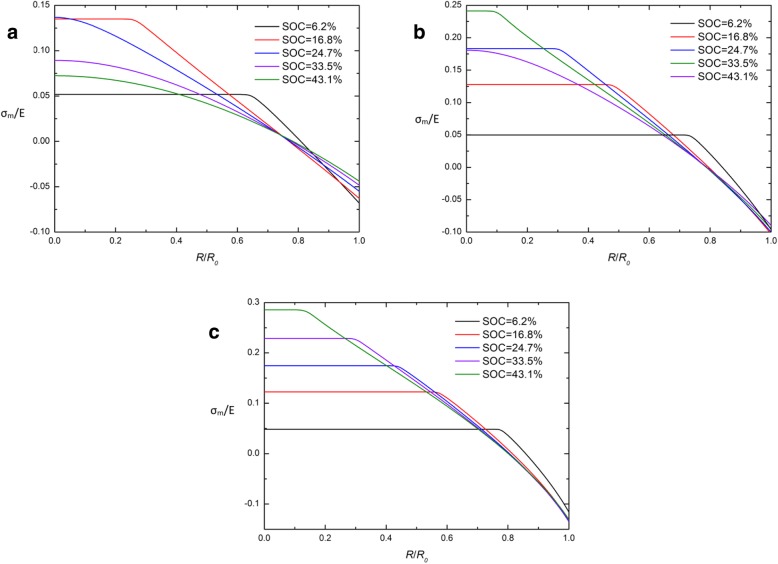
Spatial distribution of *σ*_m_/E in a spherical Si nanoparticle electrode at different SOCs under galvanostatic operation with the developed expression of chemical potential (**a**) *j* = 0.5*j*_0_, (**b**) *j* = *j*_0_, and (**c**) *j* = 1.5*j*_0_s

By comparing the traditional and developed chemical potentials in Eq. (26) and Eq. (29), it is found that the Cauchy hydrostatic stress σ_m_ is the key to the difference between these two expressions. In order to investigate the causes of CIR, the spatial distribution of σ_m_/E in a spherical Si electrode at different SOCs under galvanostatic operation with different expressions of chemical potential are given in Fig. 5 and Fig. 6. Obviously, nearly all curves intersect at one point in CIR and the Cauchy hydrostatic stress σ_m_ is close to 0 in this point, except at the beginning of charging (SOC = 6.2%). It indicates that σ_m_ in CIR is kept at a low level (nearly 0) for most of the charge period. It can be interpreted that the two chemical potential expressions are nearly equivalent when the hydrostatic stress σ_m_ is close to 0. This may partly explain why CIR appears, but it is not enough to explain the features of curves on σ_m_. It needs to be solved by our next research.

## Conclusions

In this work, a developed expression of chemical potential is proposed without the low deformation rate assumption, distinguished from the developed expression which is widely used at present. The difference between the traditional and developed expressions of chemical potential on the distributions of stress and concentration in Si nanoparticle electrodes is discussed under both potentiostatic and galvanostatic operations.

The result reveals that the effect caused by different expressions of chemical potential can be neglected under galvanostatic operation, but it is significant under potentiostatic operation. It is found that the effect is just related to the deformation rate rather than the deformation, and it can be greater with the increase of deformation rate. Considering the low deformation rate assumption in the traditional chemical potential expression, it is believed that the results obtained by the developed chemical potential expression are more reliable. A chemical potential independent region (CIR), where the flux caused by traditional and developed chemical potential is almost the same during most of the lithiated process, is found near the nanoparticle electrode surface. In addition, CIR is closer to the surface with the increases of charge current. A similar phenomenon also appears in the Cauchy hydrostatic stress curves. The Cauchy hydrostatic stress σ_m_ keeps a constant and maintained at a low level (nearly 0) in CIR at the most time, no matter which chemical potential expression is used. Such results have not yet been reported in the literature.

## Data Availability

The datasets analyzed during the current study are available from the corresponding author on reasonable request.

## Abbreviations

CIR: A region where the diffusion flux caused by these two expressions of chemical potential is nearly same
